# Single copy nuclear gene analysis of polyploidy in wild potatoes (*Solanum* section *Petota*)

**DOI:** 10.1186/1471-2148-12-70

**Published:** 2012-05-24

**Authors:** Danying Cai, Flor Rodríguez, Yuanwen Teng, Cécile Ané, Meredith Bonierbale, Lukas A Mueller, David M Spooner

**Affiliations:** 1 Department of Horticulture, the State Agricultural Ministry Key Laboratory of Horticultural Plant Growth, Development and Quality Improvement, 866 Yuhangtang Road, Zhejiang University, Hangzhou, Zhejiang, 310058, China; 2 Department of Horticulture, USDA, Agricultural Research Service, University of Wisconsin, 1575 Linden Drive, Madison, WI, 53706-1590, USA; 3 Centro Regional de Investigación Remehue, INIA, Xa Región de los Lagos, Km 8 Norte, Ruta 5 Sur, Casilla de Correos 24-O, Osorno, Chile; 4 Departments of Botany and of Statistics, 1300 University Ave., University of Wisconsin-Madison, Madison, WI, 53706-1590, USA; 5 International Potato Center, P.O. Box 1558, Lima, 12, Peru; 6 Boyce Thompson Institute for Plant Research, Tower Road, Ithaca, NY, 14853, USA

## Abstract

**Background:**

Recent genomic studies have drastically altered our knowledge of polyploid evolution. Wild potatoes (*Solanum* section *Petota*) are a highly diverse and economically important group of about 100 species widely distributed throughout the Americas. Thirty-six percent of the species in section *Petota* are polyploid or with diploid and polyploid cytotypes. However, the group is poorly understood at the genomic level and the series is ideal to study polyploid evolution. Two separate studies using the nuclear orthologs GBSSI and nitrate reductase confirmed prior hypotheses of polyploid origins in potato and have shown new origins not proposed before. These studies have been limited, however, by the use of few accessions per polyploid species and by low taxonomic resolution, providing clade-specific, but not species-specific origins within clades. The purpose of the present study is to use six nuclear orthologs, within 54 accessions of 11 polyploid species, 34 accessions of 29 diploid species of section *Petota* representing their putative progenitors, and two outgroups, to see if phenomena typical of other polyploid groups occur within wild potatoes, to include multiple origins, loss of alleles, or gain of new alleles.

**Results:**

Our results increase resolution within clades, giving better ideas of diploid progenitors, and show unexpected complexity of allele sharing within clades. While some species have little diversity among accessions and concur with the GBSSI and nitrate reductase results, such as *S. agrimonifolium*, *S. colombianum*, *S. hjertingii*, and *S. moscopanum*, the results give much better resolution of species-specific progenitors. Seven other species, however, show variant patterns of allele distributions suggesting multiple origins and allele loss. Complex three-genome origins are supported for *S. hougasii*, and *S. schenckii*, and one of the ten accessions of *S. stoloniferum*. A very unexpected shared presence of alleles occurs within one clade of *S. verrucosum* from Central America, and *S. berthaultii* from South America in six polyploid species *S. demissum*, *S. hjertingii*, *S. hougasii*, *S. iopetalum*, *S. schenckii*, and *S. stoloniferum*.

**Conclusions:**

Our results document considerable genomic complexity of some wild potato polyploids. These can be explained by multiple hybrid origins and allele losses that provide a clear biological explanation for the taxonomic complexity in wild potato polyploids. These results are of theoretical and practical benefit to potato breeders, and add to a growing body of evidence showing considerable complexity in polyploid plants in general.

## Background

### Biology of polyploids

Over 70% of the monocots [1] and 70-80% of the dicots [2] were estimated to be polyploid. Recent data from genomics, however, suggest that almost all angiosperms, perhaps even all plant groups, have experienced one to several rounds of genome duplication [3,4], sometimes followed by genome reorganization, homoeolog loss, and diploidization [3,5-12].

Polyploids are usually defined as autopolyploid (genome doubling from single species) or allopolyploid (genome doubling after hybridization or genome unreduced before hybridization) [13]. Others use the terms polysomic polyploid (instead of autopolyploid) and disomic polyploid (instead of allopolyploid) [14] to describe the genetic behavior of the plants rather than assuming anything about their origin or genome constitution.

A number of studies have focused on the single vs. multiple events of origins of polyploids [5,15-21]. Multiple origins could generate more genetic diversity in the polyploid plant species than their diploid ancestors [22-24]. This study focuses on polyploid evolution in wild potatoes (*Solanum* L. section *Petota* Dumort.).

### *Taxonomy and biology of* Solanum *section* Petota

Section *Petota* contains four cultivated species [25,26], and about 100 wild species relatives [27]. About 70% of the wild species are diploid (2n = 2x = 24), with the rest tetraploid (2n = 4x = 48) and hexaploid (2n = 6x = 72), with a few triploid or pentaploid populations [28]. The polyploids range from allopolyploids to autopolyploids. Using the classical taxonomic system of Hawkes [29] (Table 1), four taxonomic series of wild species that are wholly or predominately polyploid: series *Acaulia* (4x, 6x), *Conicibaccata* (2x, 4x, 6x), *Demissa* (6x), and *Longipedicellata* (4x). Other series with polyploids are predominately diploid: *Bulbocastana* (2x, 3x), *Commersoniana* (2x, 3x), *Maglia* (2x, 3x), *Pinnatisecta* (2x, 3x), *Piurana* (2x, 4x), and *Tuberosa* (2x, 4x, 6x) [28].

**Table 1:** Germplasm examined grouped by their classic series affiliations (Hawkes, 1990), country and state (or province or department) of collection, ploidy and endosperm balance numbers (EBN), and genome affiliations

**Section, series (ser. abbreviation)**	**Species**	**PI**	**Country, State**	**Ploidy**	**EBN**	**Genomes (Matsubayashi 1991)**	**Genomes (Hawkes 1990)**
**Polyploids**							
*Acaulia* Juz. (ACA)	*Solanum acaule* Bitter	310923	Bolivia, Cochabamba	4x	2	AAA^a^A^a^	A_2_A_2_A_3_A_3_
		472648	Argentina, Jujuy	4x	2		
		473485	Peru, Lima	4x	2		
	*S. albicans* (Ochoa) Ochoa	230494	Peru, Cajamarca	6x	4	AAA^a^A^a^XX	
		365305	Peru, Apurímac	6x	4		
		365376	Peru, La Libertad	6x	4		
		561642	Ecuador, Chimborazo	6x	4		
*Demissa* Buk. (DEM)	*S. demissum* Lindl.	161719	Mexico, Federal District	6x	4	AADDD^d^D^d^	A_1_A_1_A_4_A_4_B_1_B_1_
		225711	Columbia, Boyacá	6x	4		
		275206	Mexico, Chihuahua	6x	4		
		275211	Guatemala, Huehuetenango	6x	4		
		498012	Mexico, Durango	6x	4		
		545763	Mexico, Oaxaca	6x	4		
		558482	Mexico, Mexico	6x	4		
	*S. hougasii* Correll	161174	Mexico, Michoacán	6x	4		A_1_A_1_A_4_A_4_B_2_B_2_
		161726	Mexico, Jalisco	6x	4		
		239423	Mexico, Michoacán	6x	4		
		558402	Mexico, Jalisco	6x	4		
		558422	Mexico, Jalisco	6x	4		
	*S. iopetalum* (Bitter) Hawkes	275181	Mexico, Puebla	6x	4	AADDD^b^D^b^ (as *S. brachycarpum*)	A_1_A_1_A_4_A_4_B_3_B_3_
		275182	Mexico, Puebla	6x	4		
		498021	Veracruz	6x	4		
		498251	Mexico, Oaxaca	6x	4		
		558405	Mexico, Michoacán	6x	4		
		558409	Mexico, México	6x	4		
		607850	Mexico, Hidalgo	6x	4		
	*S. schenckii* Bitter	275261	Mexico, Oaxaca	6x	4		A_1_A_1_A_4_A_4_B_4_B_4_
		498040	Mexico, Queretaro	6x	4		
		498250	Mexico, Oaxaca	6x	4		
		545733	Mexico, Puebla	6x	4		
		558456	Mexico, Oaxaca	6x	4		
		558458	Mexico, Puebla	6x	4		
*Longipedicellata* Buk. (LON)	*S. hjertingii* Hawkes	186559	Mexico, Coahuila	4x	2		
		251067	Mexico, Nuevo León	4x	2		
		498019	Mexico, Coahuila	4x	2		
		498050	Mexico, San Luis Potosí	4x	2		
		545713	Mexico, Coahuila	4x	2		
		570625	Mexico, San Luis Potosí	4x	2		
	*S. stoloniferum* Schltdl. and Bouchet	275252	Mexico, Oaxaca	4x	2	AABB	
		283101	Mexico, Chihuahua	4x	2		
		497994	Mexico, Chihuahua	4x	2		
		498028	Mexico, Zacatecas	4x	2		
		545740	Mexico, Durango	4x	2		
		545787	Mexico, Zacatecas	4x	2		
		558395	Mexico, Baja California Sur	4x	2		
		558453	Mexico, Jalisco	4x	2		
		558454	Mexico, Queretaro	4x	2		
		558466	Mexico, Michoacán	4x	2		
*Conicibaccata* Bitter (CON)	*S. agrimonifolium* Rydb.	243350	Guatemala, Huehuetenango	4x	2	A^c1^A^c1^C^a^C^a^	
		558372	Mexico, Chiapas	4x	2		
	*S. colombianum* Bitter	561633	Ecuador, Pichincha	4x	2		
		583325	Venezuela, Táchira	4x	2		
	*S. moscopanum* Hawkes	567812	Ecuador, Loja	6x	4		
		567843	Cauca Colombia	6x	4		
**Diploids**							
*Bulbocastana* Rydb. Hawkes (BUL)	*S. bulbocastanum* Dunal	347757	Mexico, Michoacán	2x	1	A^b^A^b^	
*Conicibaccata* Bitter (CON)	*S. chomatophilum* Bitter	310991	Peru, Amazonas	2x	2	A^c2^A^c2^	
		762575	Peru, Cajamarca.	2x	2		
	*S. laxissimum* Bitter	760350/473372	Peru, Cuzco	2x	2		
	*S. limbaniense* Ochoa	762336	Peru, Puno	2x	2		
		762848/607887	Peru, Cuzco	2x	2		
	*S. violaceimarmoratum* Bitter	473396	Bolivia, Cochabamba	2x	2		
		761797	Peru, Cuzco	2x	2		
		760331	Bolivia, La Paz.	2x	2		
*Cuneoalata* Hawkes (CUN)	*S. infundibuliforme* Phil.	472857	Argentina, Jujuy	2x	2	AA	
*Lignicaulia* Hawkes (LIG)	*S. lignicaule* Vargas	473351	Peru, Cuzco	2x	1		
*Megistacroloba* Cárdenas and Hawkes (MEG)	*S. boliviense* Dunal	597736	Bolivia, Potosí	2x	2	AA	
	*S. raphanifolium* Cardenas & Hawkes	265862	Peru, Cuzco	2x	2	AA	
*Pinnatisecta* (Rydb.) Hawkes (PIN)	*S. trifidum* Correll	255536	Mexico, Michoacán	2x	1		
	*S. stenophyllidium* Bitter	255527	Mexico, Aguascalientes	2x	1		
*Piurana* Hawkes (PIU)	*S. albornozii* Correll	498206	Ecuador, Loja	2x	2		
	*S. cantense* Ochoa	762130	Peru, Lima	2x	2		
	*S. chilliasense* Ochoa	761590	Ecuador, El Oro	2x	2		
	*S. chiquidenum* Ochoa	762950	Peru, Julcán	2x	2		
	*S. hypacrarthrum* Bitter	761259	Peru, Ancash	2x	1		
	*S. piurae* Bitter	761072	Peru, Piura	2x	2	A^P^	
*Polyadenia* Correll (POL)	*S. polyadenium* Greenm.	161728	Bolivia, Michoacán	2x	untested, likely 1	A^po^A^po^	
*Tuberosa* (Rydb.) Hawkes (TUB)	*S. andreanum* Baker	320345	Colombia, Cauca	2x	2		A_1_A_1_
	*S. brevicaule* Bitter	498091	Bolivia, Santa Cruz	2x	untested, likely 2		
	*S. berthaultii* Hawkes	265857	Bolivia, Cochabamba	2x	2		A_1_A_1_
	*S. brevicaule* Bitter	310957	Peru, Cuzco	2x	2	AA	A_1_A_1_
		498115	Cochabamba	2x	2		A_1_A_1_
	*S. candolleanum P.* Berthault	266385	Peru, Junin	2x	2	AA	A_1_A_1_
	*S. cajamarquense* Ochoa	310988	Peru, Cajamarca	2x	1		
	*S. gandarillasii* Cárdenas	265866	Bolivia, Cochabamba	2x	2		A_1_A_1_
	*S. microdontum* Bitter	500036	Argentina, Salta	2x	2	AA	A_1_A_1_
	*S. verrucosum* Schltdl.	161173	Mexico, Michoacán	2x	2	AA	A_1_A_1_
*Yungasensa* Correll (YNG)	*S. chacoense* Bitter	275138	Argentina, Tucumán	2x	2		
	*S. berthaultii* Hawkes	442689	Argentina, Salta	2x	2		
Outgroup	*S. etuberosum* Lindl.	498311	Chile, Bio-Bio	2x	1	E^e^E^e^	
Outgroup	*S. dulcamara*	Spooner 2988	USA, Wisconsin	/	/		

The origin of potato polyploids has been the subject of much debate, incorporating data from crossing studies, cytogenetics, morphology, and biogeography. Hawkes [29] speculated that section *Petota* arose in North and Central America, and possessed white stellate corollas, B genomes, and endosperm balance numbers (EBN) of 1. EBN is a strong biological isolating mechanism, empirically determined through artificial interspecific crosses and cytological examinations, and evidenced by endosperm death in EBN incompatible crosses. Entirely on the basis of empirical data, *Solanum* species have been assigned EBN based on their ability to hybridize with each other [30]. Barring other crossing barriers, successful hybridization is expected when male and female gametes have matching EBN, regardless of ploidy. Ploidy(EBN) combinations in potato include 2*x*(1EBN), 2*x*(2EBN), 4*x*(2EBN), 4*x*(4EBN), and 6*x*(4EBN). Hawkes [29] speculated that some of the North and Central American 2x(1EBN) species migrated to South America with A genome, rotate corollas and 2 or 4 EBN. A return migration of A genome back to Mexico and Central America took place around 3.5 MA, followed by polyploid formation of series *ConicibaccataDemissa*, and *Longipedicellata* with rotate corollas.

Molecular phylogenetics suggests a classification of *Solanum* section *Petota*, and of polyploid origins in potato, often quite at odds with traditional classifications as outlined by section and series affiliations [29] (Table 1). The latest comprehensive taxonomic treatment of the section by Hawkes [29] recognized 232 tuber-bearing and non-tuber-bearing species divided into 21 taxonomic series. Plastid DNA restriction site studies [31], and nuclear DNA sequencing studies [32-37] have greatly changed our understanding of ingroup relationships in section *Petota*. Relative to the last comprehensive taxonomic treatment by Hawkes [29], section *Petota* now excludes the non-tuber-bearing species, reclassified as sections *Etuberosum* (Bukasov and Kameraz) A. Child, *Juglandifolia* (Rydberg) A. Child, and *Lycopersicoides* (A. Child) Peralta [38]. Many of Hawkes 21 series are shown to be unnatural and the tuber-bearing species are divided into four clades (1 – 4) based on plastid DNA restriction site data or three clades based on nuclear DNA sequencing data, with both results similar except that the nuclear DNA sequencing data fail to distinguish clades 1 and 2. To maintain correspondence with the prior literature, we therefore refer to the nuclear clades here, as in prior studies, as clades 1 + 2, 3, 4. The allopolyploids combine sequences from different clades.

At lower taxonomic levels and important to this study, the Mexican hexaploid species *S*. *demissum* was shown to be related to the South American tetraploid species *S*. *acaule* and *S*. *albicans*, not to other members of series *Demissa*[39-41]. Spooner et al. [42] used these results to classify *S*. *acauleS*. *albicans*, and *S*. *demissum* in an informal Acaulia group, and the other members of series *Demissa* (*S*. *hougasiiS*. *iopetalum*, and *S*. *schenckii*) in an informal Iopetala group. Because of complex hybrid origins and allopolyploid origins they also used the terms Conicibaccata group and Longipedicellata group instead of series. We use the terms Acaulia, Conicibaccata, Iopetala, and Longipedicellata groups in the text as they are putatively more natural, but show Hawkes’s [29] traditional series classifications in Table 1.

The genome constitution of potato polyploids has been investigated by various workers [32, 33, 43, 44; Table 1. Matsubayashi [43] speculated on the genome formation of diploid and polyploid species via insights from cytological analysis. *Solanum acaule* was thought to be a segmental allotetraploid with minor variants of a common A genome. *Solanum agrimonifoliumS*. *colombianum*; and *S*. *stoloniferum* were designated as strict allotetraploids, and shared the same A genome with *S*. *verrucosum*, the sole A genome species from Mexico. *Solanum demissum* was thought to be an allohexaploid with two similar genomes and a third different genome, which also had one common genome with many of diploid Conicibaccata group and series *Megistacroloba* and *Tuberosa* (Table 1). Pendinen et al. [44] supported a genome constitution of AABB for *S*. *hjertingii* and *S*. *stoloniferum* with genomic in situ hybridization (GISH) analysis, and they proposed *S*. *verrucosum* for the A genome donor, as well as at least one of three species in series *Pinnatisecta* (*S*. *cardiophyllumS*. *ehrenbergii*, or *S*. *jamesii*) for the B genome donor, in concordance with the phylogenetic results using nuclear ortholog DNA sequences [32,33]. Nuclear DNA sequencing studies [32-36] supported clade 1 + 2 to contain B genome species, clade 3 to contain P genome species, and clade 4 to contain A genome species.

Phylogenetic studies in section *Petota* have been hindered by the use of single genes with insufficient data to construct well resolved phylogenies. Recently, multiple nuclear orthologs have been shown to be phylogenetically useful across different angiosperm clades. Wu et al. [45] published a set of conserved orthologous nuclear markers that they termed conserved orthologous set II markers that provided superior phylogenetic resolution in *Solanum*[34-36,46]. We use these markers in our study but use the simple term nuclear orthologs because all low copy nuclear orthologous genes are similar, requiring about the same level of care concerning technical issues (e.g. PCR recombination) and are subjected to the same set of lineage-specific and hence variable evolutionary properties (variation in rates, degree of gene conversion, gene amplification or loss).

Cloning was the traditional approach for uncovering allelic variants in allopolyploids in these nuclear ortholog allopolyploid studies, but this technique is hindered by the formation of chimeric sequences combining the sequences of different alleles [47], high labor, and high cost [48]. Consequently, our study stimulated us to develop single strand conformation polymorphism (SSCP) that separates alleles by their different physical conformations, not by size, alleviating all three of these problems [49]. Asymmetric PCR single-strand conformation polymorphism is an efficient alternative technique for isolating allelic variants of highly heterozygous individuals that eliminates two common problems encountered in cloning: PCR recombination and heteroduplex fixation. It works by the electrophoretic separation of single-stranded nucleic acid, with differing tertiary structures formed by sequence differences as small as a single base pair, with visual detection using biological stains or radioactivity.

The present research is an outgrowth of our prior DNA phylogenetic [32,33,35,36], and GISH [44] studies of polyploidy in section *Petota*, and use of nuclear ortholog markers for phylogenetic studies as we used in diploids [34]. We pose the following questions: 1) Do individual species of wild polyploid potatoes have single or multiple origins? 2) Which one of several diploid parents is the possible progenitor of the polyploids? 3) How do the results of nuclear ortholog sequences compare to prior results using the nuclear orthologs GBSSI and nitrate reductase?

## Results

### Sequence alignment and variation

The aligned length of the individual six nuclear orthologs ranged from 461 characters for C2At1g32130 to 1473 for C2At1g20050. Introns were present in all of them, but these posed no particular alignment difficulties. The total aligned length of all six nuclear orthologs was 4719 characters, although as described below we did not use a concatenated dataset. The sequence data are deposited in GenBank (Table 2) and the aligned matrix is available in TreeBASE (http://www.treebase.org) Study Accession URL: http://purl.org/phylo/treebase/phylows/study/TB2:S12288.

**Table 2:** GenBank numbers for the nuclear ortholog DNA sequence data used in this study

**Nuclear ortholog markers**	**GenBank numbers**
C2At1g32130	FJ599275-FJ599284; HQ642429 - HQ642598
C2At5g14320	FJ599363-FJ599372; HQ642599 - HQ642764
C2At1g13380	FJ599242-FJ599251; HQ642096 - HQ642267
C2At1g20050	FJ599264-FJ599273; HQ642268 - HQ642428
C2At5g47390	HQ641924 - HQ642095
C2At4g10050	HQ641753 - HQ641923

### Phylogenetic analysis of the diploids

**Figure 1:**
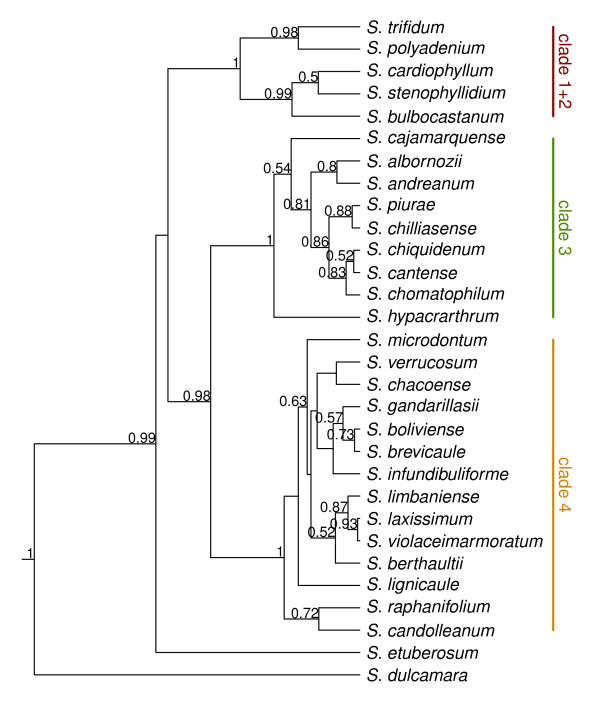
Bayesian estimate of the diploid species tree using six COS and the coalescent-based program *BEAST. Clades posterior probabilities above 0.50 are indicated above edges.

*BEAST recovered three clades for the diploid species (Figure 1) with *S. dulcamara* recovered as outgroup and *S*. *etuberosum* as a close sister group of section *Petota* as in all prior nuclear DNA sequence phylogenies [32-36]. Our *BEAST results using all six nuclear orthologs placed clades 3 and 4 as sister, with 1 + 2 sister to clade (3 + 4), as in two other nuclear ortholog phylogenies [34,45]. A GBSSI study [32] placed these three clades as polytomies, but another GBSSI study [37] and a nuclear ortholog study placed clades 1 + 2 and 4 as sister. These results clearly define these three clades, but the relationships among them are ambiguous. Because we used more sequence data here than in [32,33] and because of the concordance of our present results with [34,35], we consider the present cladistic structure to represent a dominant phylogeny. Not all prior analyses used the same species, but our study resolved all species at least in clade 4, although with low posterior probabilities for some relationships.

### Phylogenetic results of the polyploids

**Figure 2:**
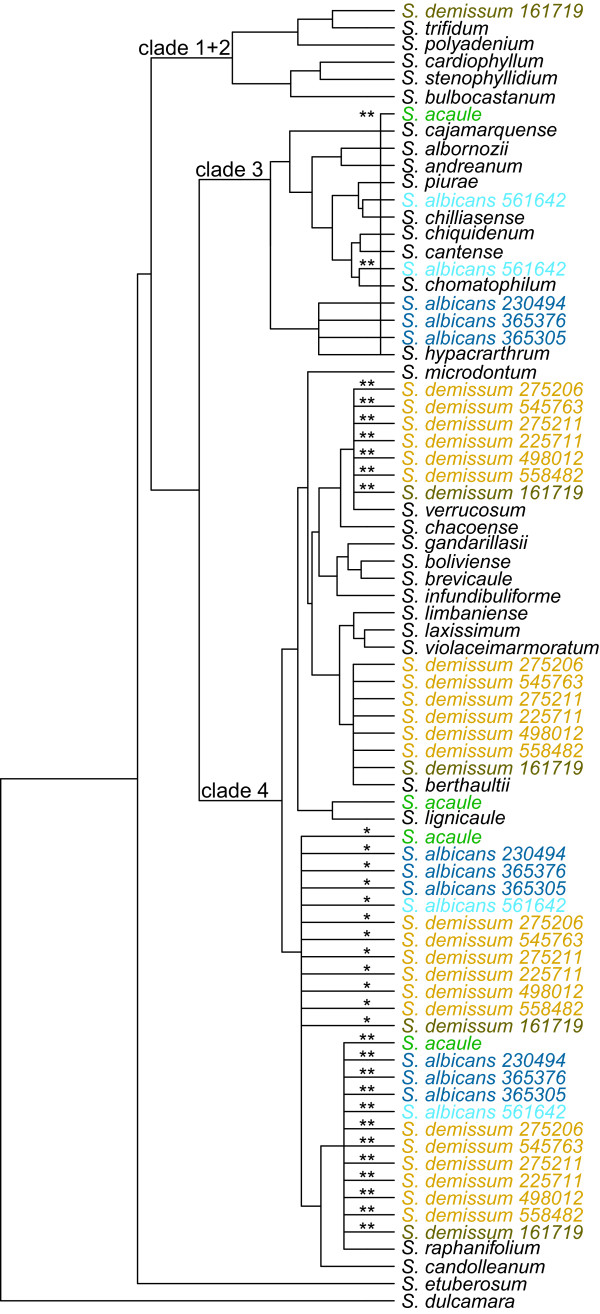
Tree summarizing the results of individual-allele analyses with the minimum evolution species tree (MEst) method, to place the polyploid alleles from the Acaulia group on the backbone diploid species tree from *BEAST (Figure1**).** Colors correspond to accessions and rows in Table 3. The placement of parental origins which received a bootstrap support of 70% or higher for one of more alleles are indicated. Placements with lower bootstrap support are not indicated. Stronger bootstrap support (90%-95% or 95%-100%) for the placement of one or more alleles is indicated with stars (* or **). There was evidence of at least one allele of *S. acaule* originating from within clade 3 (bootstrap support of 96%) even though no single placement could be identified with strong bootstrap support. This origin from clade 3 is indicated with a vertical bar spanning the whole clade.

**Figure 3:**
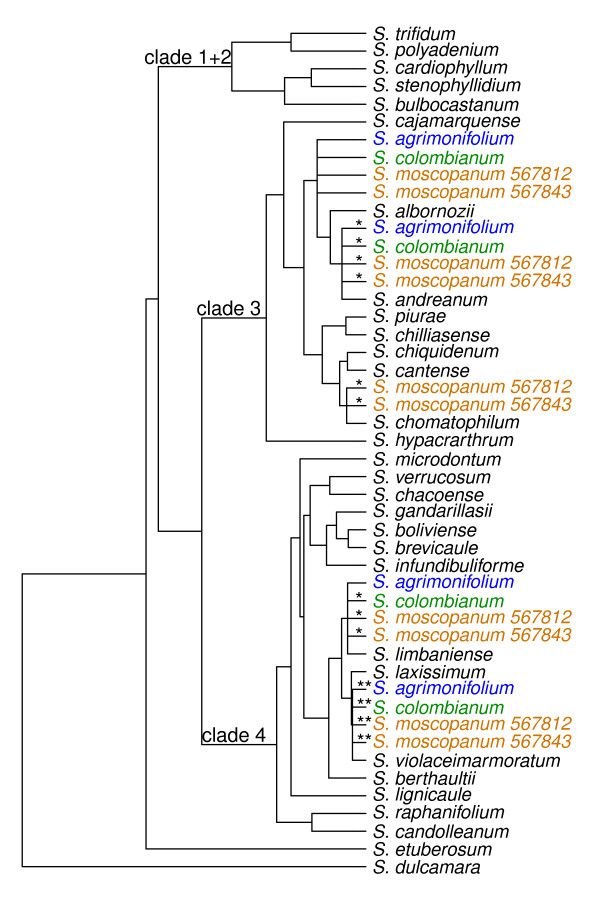
**Tree summarizing the placement of the polyploid allele origins from the Conicibaccata group on the backbone diploid species tree.** Origin placements with bootstrap support over 70% for one or more alleles are displayed, as in Figure 2.

**Figure 4:**
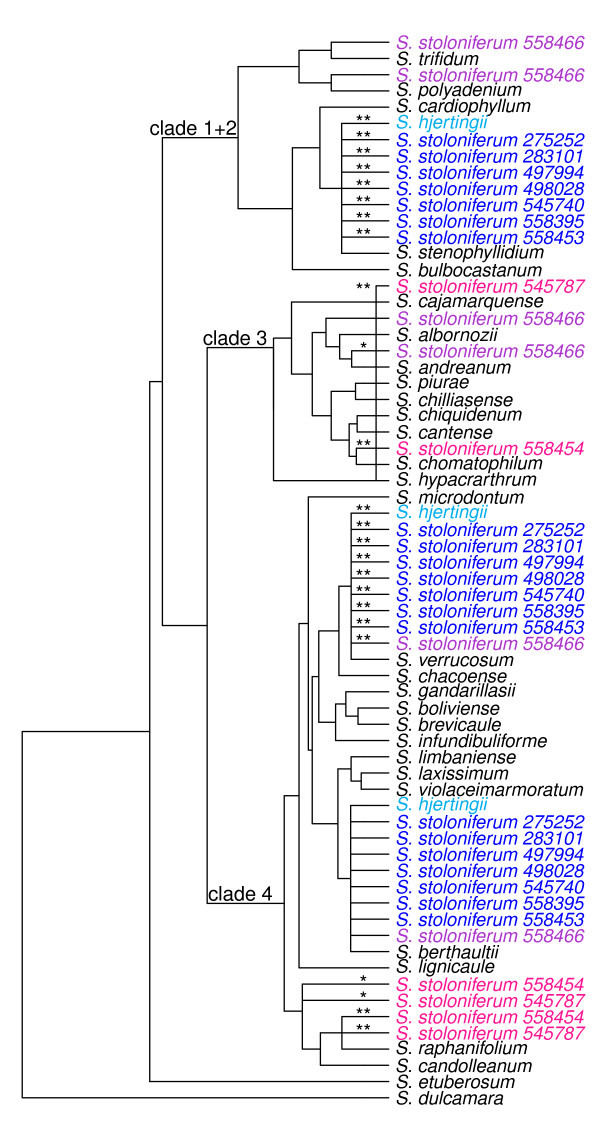
**Tree summarizing the placement of the polyploid allele origins from the Longipedicellata group on the backbone diploid species tree.** Origin placements with bootstrap support over 70% for one or more alleles are displayed, as in Figure 2. There was evidence of at least one allele of *S. stoloniferum* 545787 originating from within clade 3 (bootstrap support of 96%) even though no single placement could be identified with strong bootstrap support. This origin from clade 3 is indicated with a vertical bar spanning the whole clade.

**Figure 5:**
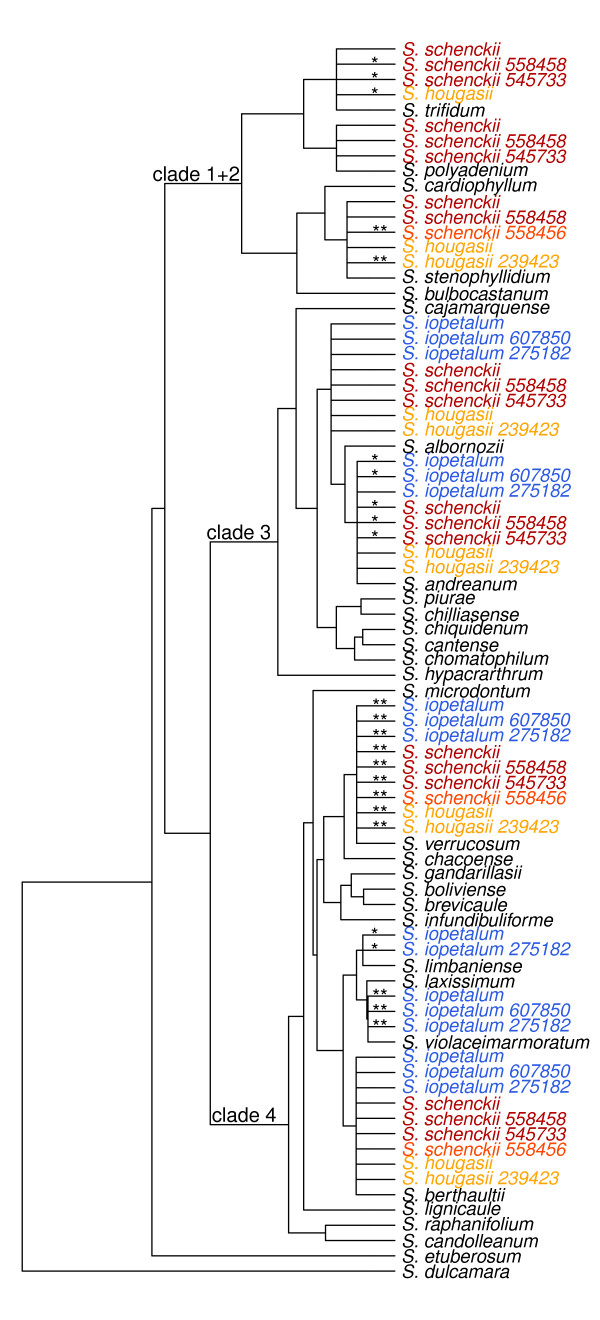
**Tree summarizing the placement of the polyploid allele origins from the Iopetala group on the backbone diploid species tree.** Origin placements with bootstrap support over 70% for one or more alleles are displayed, as in Figure 2. Tips named *S. hougasii* represent 4 of the *S. hougasii* accessions with similar results (161174, 161726, 558402, 558422). Similarly, tips marked *S. iopetalum* represent 5 of the *S. iopetalum* accessions (275181, 498021, 498251, 558405, 558409) and tips marked *S. schenckii* represent 3 of the *S. schenckii* accessions (275261, 498040, 498250).

We analyzed all 54 of our polyploid accessions separately (Table 1, Figures 2, 3, 4 and 5). Four tetraploid species,*S. acaule* (three accessions examined), *S. agrimonifolium* (2), *S. colombianum* (2), and *S. hjertingii* (6) had invariant phylogenetic results among accessions within species, and *S. agrimonifolium* and *S. colombianum* had identical results to each other. The origin of some alleles is supported by high bootstrap values at the species level, which we report below and map in Figures 2, 3, 4 and 5 (Table 3). Other origin placements are ambiguous at the species level but well supported at the clade level.

**Table 3:** **Summary of cladistic relationships of the polyploid alleles that are highly supported at the species level (mapped in Figures**2**,**3**,**4**and**5**)**

**Species**	**Number of accessions/species examined**	**Clade 1 + 2**	**Clade 3**	**Clade 4**
*Solanum acaule*	310923, 472648, 473485	No allele from clade 1 + 2 with BS = 0.85	Precise origin not identified, but at least 1 allele with BS = 0.96.	*S. lignicaule; S. candolleanum and S. raphanifolium clade*
*S. albicans*	230494, 365305, 365376	No allele with BS ≥ 0.70	*S. hypacrarthrum*	*S. candolleanum and S. raphanifolium clade*
	561642	No allele with BS = 0.84	*S. chilliasense* and *S. piurae clade; S. chomatophilum*	*S. candolleanum and S. raphanifolium clade*
*S. demissum*	225711, 275206, 275211, 498012, 545763, 558482	No allele with BS ≥ 0.82	Precise origin not identified, but at least 1 allele with BS > 0.96 for all accessions	*S. berthaultii; S. verrucosum; S. candolleanum and S. raphanifolium clade*
	161719	*S. trifidum*	Precise origin not identified, but at least 1 allele with BS = 0.95	*S. berthaultii, S. verrucosum, S. candolleanum and S. raphanifolium clade*
*S. agrimonifolium, S. colombianum*	243350, 558372, 561633, 583325	No allele with BS ≥ 0.91	*S. andreanum* and *S. albornozii clade*	*S. laxissimum, S. limbaniense, and S. violaceimarmoratum clade*
*S. moscopanum*	567812, 567843	No allele with BS ≥ 0.92	*S. andreanum* and *S. albornozii clade; S. chomatophilum*	*S. laxissimum, S. limbaniense, and S. violaceimarmoratum clade*
*S. hougasii*	161174, 161726, 558402, 558422, 239423	S. stenophyllidium; S. trifidum (ambiguous for 239423)	*S. andreanum* and *S. albornozii clade*	*S. berthaultii, S. verrucosum*
*S. iopetalum*	275181, 275182, 498021, 498251, 558405, 558409, 607850	No allele with BS ≥ 0.90	*S. andreanum* and *S. albornozii clade*	*S. berthaultii, S. verrucosum; S. laxissimum, S. limbaniense, and S. violaceimarmoratum clade*
*S. schenckii*	275261, 498040, 498250, 558458,	S. polyadenium; S. stenophyllidium; S. trifidum	*S. andreanum* and *S. albornozii clade*	*S. berthaultii; S. verrucosum*
	545733	S. polyadenium; S. trifidum	*S. andreanum* and *S. albornozii clade*	*S. berthaultii; S. verrucosum*
	558456	S. stenophyllidium	No allele from clade 3 with BS = 0.99	*S. berthaultii; S. verrucosum*
*S. hjertingii*	186559, 251067, 498019, 498050, 545713, 570625	S. stenophyllidium	No allele with BS ≥ 0.84	*S. berthaultii; S. verrucosum*
*S. stoloniferum*	275252, 283101, 497994, 498028, 545740, 558395, 558453		No allele with BS ≥ 0.84	*S. berthaultii; S. verrucosum*
*S. stoloniferum*	558454, 545787	No allele with BS ≥ 0.83	*S. chomatophilum* (species origin within the clade ambiguous for 545787)	*S. candolleanum* and *S. raphanifolium clade*
*S. stoloniferum*	558466	*S. polyadenium, S. trifidum*	*S. andreanum* and *S. albornozii* clade	*S. berthaultii; S. verrucosum*

The origin placements that are ambiguous at the species level but well supported at the clade level are also indicated.

### Acaulia group (tetraploid and hexaploid)

*Solanum acaule* (tetraploid) shows distribution of most of its alleles very closely related within clade 4, with some of them placed confidently as sister to *S. lignicaule* and the *S. candolleanum* and *S. raphanifolium* clade (Figure 2). Some of its alleles also fall within clade 3. The hexaploid species *S. albicans*, has three of the four accessions sharing alleles with the *S. raphanifolium* and *S. candolleanum* clade (but not with *S. lignicaule*), and an additional allele with *S. hypacrarthrum* in clade 3. The remaining accession, PI 561642, shares alleles with the *S. raphanifolium* and *S. candolleanum* clade, but shares clade 3 alleles with *S. chilliasense* and *S. chomatophilum*, not with *S. hypacrarthrum*. The hexaploid species *S. demissum* has six of its seven accessions sharing alleles with the *S. raphanifolium* and *S. candolleanum* clade, and with *S. berthaultii* and *S. verrucosum* (and with alleles in clade 3 but with ambiguous species association). The remaining accession of *S. demissum*, PI 161719, shares these same alleles but an additional allele with *S. trifidum* in clade 1 + 2.

### Conicibaccata group (tetraploid and hexaploid)

All accessions of *S*. *agrimonifolium* and *S. colombianum* (tetraploid) are identical, grouping with all other examined diploid members of this series (*S. laxissimum*, *S. limbaniense*, *S. violaceimarmoratum*) in clade 4, and with the *S. albornozii* and *S. andreanum* clade in clade 3 (Figure 3). The two examined accessions of *S. moscopanum* (hexaploid) have identical clade associations to *S. agrimonifolium* and *S. colombianum*, but with an additional allele with *S. chomatophilum*.

### Longipedicellata group (all tetraploid)

All six accessions of *S. hjertingii*, and seven of the ten accession of *S. stoloniferum* share alleles with *S. berthaultii* and *S. verrucosum* in clade 4, and an allele with *S. stenophyllidium* in clade 1 + 2 (Figure 4). The three remaining accessions of *S. stoloniferum* are all different from these accessions. One accession of *S. stoloniferum*, PI 558466, is identical to the above regarding clade 4, but shares alleles with the *S. polyadenium* and *S. trifidium* clade of clade 1 + 2, not with *S. stenophyllidium*, and alleles with the *S. andreanum* and *S. albornozii* clade of clade 3. The remaining accessions of *S. stoloniferum*, PI 558454 and PI 545787, share alleles with *S. candolleanum* and *S. raphanifolium* of clade 4 and alleles on clade 3.

### Iopetala group (all hexaploid)

All seven accessions of *S. iopetalum* share alleles with three clades of clade 4, *S. berthaultii*, *S. verrucosum*, and the three species of the diploid series Conicibaccata clade (*S. laxissimum*, *S. limbaniense*, and *S. violaceimarmoratum*). In addition, they share alleles with the *S. andreanum* and *S. albornozii* clade of clade 3 (Figure 5). Five of the six accessions of *S. schenckii* share alleles with *S. berthaultii* and separately *S. verrucosum* of clade 4, with the *S. andreanum* and *S. albornozii* clade of clade 3, and three separate clades in clade 1 + 2, *S. polyadenium*, *S. trifidum,* and *S. stenophyllidium* (although with low support for accession PI 545733). The remaining accession of *S. schenckii*, PI 558456, shares alleles with *S. berthaultii* and *S. verrucosum* of clade 4, and with *S. stenophyllidium* of clade 1 + 2, but lacks alleles in clade 3.

All five accessions of *S. hougasii* are identical to four of six accessions of *S. schenckii* regarding alleles in clades 3 and 4, but *S. schenckii* shares alleles with three species (*S. polyadenium*, *S. stenophyllidium*, *S. trifidum*) in clade 1 + 2, and *S. hougasii* two species (*S. stenophyllidium*, *S. trifidum*).

## Discussion

### Phylogenetic results concordant with prior single-copy GBSSI and nitrate reductase results but with better resolution within clades

The diploid tree (Figure 1) recovers all three nuclear clades (1 + 2, 3, 4) concordant with prior results mentioned in Background. Forty-four of the 54 accessions in our present study place alleles in the major clades concordant with results from GSSSI [32] and nitrate reductase [33]. These include *S. acaule* (3 of 3 accessions), *S. agrimonifolium* (2 of 2), *S. colombianum* (2 of 2), *S. demissum* (6 of 7), *S. hjertingii* (6 of 6), *S. hougasii* (5 of 5), *S. iopetalum* (7 of 7), *S. moscopanum* (2 of 2), *S. stoloniferum* (7 of 10), and *S. schenckii* (4 of 6) (Table 3).

However, our results provide much greater resolution of species-specific associations of polyploid alleles within these clades. For example, one recurrent result is the distribution of polyploid alleles between the geographically separate clade 4 species *S. verrucosum* (Mexico) and *S. berthaultii* (central South America), as found in the North and Central American polyploids *S. demissumS. hougasiiS. iopetalumS. hjertingiiS. stoloniferum*, and *S. schenckii*. One possible explanation arises from the biogeographic hypothesis of Hawkes [29] who postulates that *S. verrucosum* evolved from a species that migrated from South America to Mexico, and was the A-genome (Table 1) contributor to these North and Central American polyploids. *Solanum berthaultii* (or its close relative) could have been that South American species, and *S. verrucosum* could retain some of its alleles. Another possible interpretation could be statistical error due to a violation of the coalescent model used by the species tree methods used here, such as gene flow across different species.

### Phylogenetic results incongruent with prior single-copy GBSSI and nitrate reductase results

Ten of the remaining 54 accessions show missing alleles or alleles in new clades 1 + 2, 3, 4, relative to the prior single-copy nuclear phylogenies [32,33] (Table 3). One example of new alleles relative to prior studies is found in *S. albicans* (hexaploid) that is morphologically very similar to one of its putative parents, *S. acaule*. Both species are cytological allopolyploids and the origin of the third genome in *S. albicans* relative to *S. acaule* has been the subject of long investigation [50]; note its unknown nature as designated by XX by Matsubayashi [43] (Table 1). While the nuclear RFLP study of Hosaka and Spooner [50] could distinguish the genetic difference between *S. acaule* and *S. albicans*, no clade 3 species were used, and no clade 3 alleles were found in the single nuclear gene studies of [32,33]. This is the first study that documents clade 3 genomes in *S. albicans*. Of great interest is that of the four examined accessions of this species, one of them, PI 561642, is a northern disjunct in central Ecuador, the others all distributed in central to northern Peru. Based on AFLP and morphological data, Kardolus [51] recognized a new subspecies of *S. acaule* subsp. *palmirense* from the very accession we examined here. Although it has the hexaploid chromosome number and overall morphological similarity to *S. albicans*, AFLP data influenced him to classify it in *S. acaule* (typically tetraploid). We recognize this accession as *S. albicans*, but show a separate clade 3 genome origin for this species.

We found great variation in *S. stoloniferum*. While seven of the ten accessions showed identical origins to each other and to all six accessions of *S. hjertingii* (both species were the sole members of the Longipedicellata group), three accessions showed very different distributions of alleles. Accessions 558454 and 545787 lacked alleles from clade 1 + 2; and shared clade 4 alleles with clade *S. candolleanum* and *S. raphanifolium* but not with *S. berthaultii* and *S. verrucosum*. Accession 558466 shared clade 3 alleles with *S. andreanum* and *S. albornozii*, and clade 4 alleles with *S. berthaultii* and *S. verrucosum*. Accessions 558454 and 558466 are the only two accessions documented with alleles in clade 3.

*Solanum schenckii* PI 545733 has the same distribution of alleles as *S. stoloniferum* PI 558466. Unlike four of the six accessions of *S. schenckii*, it lacks alleles from *S. stenophyllidium*. *Solanum schenckii* PI 558456 is the only accession of this species that lacks alleles from clade 3. *Solanum demissum* PI 161719 has clade 4 alleles shared with all other 6 examined members of this species, but in addition possesses an allele from clade 1 + 2.

Various processes could explain the results we found above, to include multiple origins, introgressive hybridization subsequent to speciation, allele losses, or in the case of apparent allele losses because of procedural errors that failed to sequence “missing” alleles. We attempted to avoid the latter error, however, by our use of SSCP and cloning when expected alleles could not be located (Methods).

Multiple origins of polyploids appear to be a recurring and common pattern in plants. They have been documented in groups as diverse as the angiosperms in the Araliaceae [52], Asteraceae [24,53], Brassicaceae [54], Leguminosae [55,56], and Saxifragaceae [17]; in the bryophytes [57]; and ferns [22]. Regarding “missing” alleles, we consider missing alleles to be real, rather than an artifact of poor procedure, because of the procedures we outline in Methods. Allele loss in polyploids is appearing to be a common pattern in other groups [11]. Its cause could be stochastic, or caused by “genomic shock” during the early stages of polyploid formation [58]. Genomic changes are believed to be more common in allopolyploids than in autopolyploids, possibly correlated with greater genomic shock expected in genomically divergent parents of allopolyploids relative to diploids. The only study of genetic changes in section *Petota* was conducted in a synthetic autopolyploid [59], and this showed fewer expression differences than has been found in many allopolyploids [60]. However, this was conducted only with a first generation hybrid and was not subjected to selective forces allowing possible genomic rearrangements, so these data have little applicability here. The majority of the polyploids studied here are presumed allopolyploids or segmental autopolypolyploids (Table 1).

### Implications for the taxonomy of polyploids

The taxonomy of *Solanum* section *Petota* (including both the cultivated and wild potato species) is complicated by sexual compatibility among many species, introgression, interspecific hybridization, auto- and allopolyploidy, a mixture of sexual and asexual reproduction, possible recent species divergence, phenotypic plasticity, and consequent great morphological similarity and difficulty in defining and identifying species [27]. As this study and others [34-36] demonstrate, it is also complicated by phylogenetic results that are often incongruent among different phylogenetic markers (in this case nuclear ortholog markers).

Polyploids have long been recognized to be complex taxonomically and to complicate species coherence [61,62]. Thirty-six percent of the species in section *Petota* are polyploid or with diploid and polyploid cytotypes [28] and section *Petota* is notably difficult taxonomically. The present study documents considerable genomic complexity in polyploids in section *Petota*, helping to explain why taxonomists have traditionally had such difficulty in providing an easy taxonomic treatment of this group. Additional studies using more accessions and nuclear orthologs surely would expand such examples. Our results provide the very practical outcome in helping explain the cause of such taxonomic complexity, guiding taxonomists and genebank managers to rational classifications that do not search for clear differences. Clear differences will likely never be found in such systems. Our results also alert breeders to a storehouse of diversity within traditionally recognized polyploid species.

### Statistical analysis with polyploids and gene tree discordance

We faced here two major difficulties for tree inference. First, the discordance among nuclear gene trees was extensive even among diploid species. Second, the presence of multiple alleles for polyploids precluded a concatenated approach. More generally, alleles from a polyploid species cannot be paired up across different genes a priori. Much recent research has been devoted to address the first issue of species tree reconstruction from multiple conflicting gene trees [63]. In contrast, there is no standard statistical method to deal with the second issue and reconstruct the reticulate history of polyploid species from multiple gene trees. Our study illustrates a novel approach to dealing with these two issues. To account for a non-tree like history of polyploidy species, each polyploidy allele was placed separately within the diploid species tree, which was inferred using a gene tree/species tree approach to account for gene tree discordance. Finally, we summarized the results from all alleles and all accessions of a polyploid species by displaying the well supported parental origins. This workflow could be applicable to many other groups of organisms for the inference of polyploidy origins, in the presence of extensive gene tree discordance.

### Future approaches

Buggs et al. [64] developed next generation sequencing approaches for investigating genomic changes in *Tragopogon* (Compositae) that highlight one possible next direction in this study, especially considering the availability of a genomic sequence for potato [65]. These investigators examined a wide range of genomic changes, including gene loss (quantifying the rapidity of such losses and examining parental biases in gene loss), gene silencing, subfunctionalization, and developing FISH markers for the study of genomic structural changes. Their general approach involves building an extensive genetic framework for the diploid parents via next-generation sequence data (using a combination of 454 and Illumina platforms), and then developing species-specific SNPs that are useful to investigate gene loss in the allopolyploids. They also used this approach to identify loci that exhibit apparent altered gene expression (silencing, or up- or down- regulation) in a selection of individuals of an allopolyploid relative to the parental alleles. A unique feature of their study was the use of Sequenom MassARRAY iPLEX genotyping to conduct a broad survey of homeolog loss across multiple allopolyploid populations. This method, which has been used in corn genomics, is especially suited for detecting homeologs that differ at only a few nucleotide positions. They make the point that next-generation sequencing technologies can be easily and inexpensively applied to many plant species, making any evolutionarily provocative system a potential new “model” system.

## Conclusions

Our results document considerable genomic complexity of some wild potato polyploids. These can be explained by multiple hybrid origins and allele losses that provide a clear biological explanation for the taxonomic complexity in wild potato polyploids. These results are of theoretical and practical benefit to potato breeders, and add to a growing body of evidence showing considerable complexity in polyploid plants in general.

## Methods

### Plant materials, DNA isolation, amplification, and SSCP band sequencing

Fifty-four polyploid accessions, using 2–10 accessions per species from the Acaulia group (4x, 6x), Conicibaccata group (4x, 6x), Longipedicellata group, (4x), and Iopetala group (6x), were examined in our study (Table 1). We also examined 34 diploid accessions of 29 diploid species containing ingroup species of section *Petota* in series *BulbocastanaCuneoalataLignicauliaMegistacrolobaPinnatisectaPiuranaPolyadeniaTuberosa* and *Yungasensa*, the Conicibaccata group, and two outgroups (*S*. *dulcamaraS*. *etuberosum*) (Table 1). These diploid species were chosen based on prior hypotheses of diploid progenitors of the polyploids [29,32,33,43,44], or results of phylogenetic studies within section *Petota*, including the polyploids [31-33,35,36]. DNA obtained from leaves of young plants grown from seeds in a greenhouse was extracted by the CTAB method [66] and qualified and quantified in 1% agarose gels with marker CsCl-purified λ DNA digested with *Pst*I. All DNA amplification, and SSCP sequencing followed [49]. In brief, SSCP involved running SSCP, extracting the bands of interest, and sequencing them. In a few cases alleles could not be separated by SSCP because of smearing or poor amplification of the PCR products and these PCR products were then cloned and sequenced as in [34]. When we failed to obtain a DNA sequence found in prior results, or in the majority of the accessions examined here, we performed SSCP twice more. For example, in tetraploids, we expected two alleles in the PAGE or MDE gel while for hexaploids we expected three alleles. If the number of the alleles in one accession were less than these, we reran them in PAGE or MDE gels to make sure the allele number was right. Sometimes, the recovered bands of those accessions with missing alleles could not be amplified in the PCR for sequencing, and in such cases we then cloned our fragments as a final check of potential missing alleles.

### Model selection

The molecular substitution models were evaluated with ModelTest [67] to select the preferred model among those that could be used in *BEAST, separately for each locus. We used a likelihood ratio test to compare nested models, with a forward step-wise approach. For all loci, the selected model accounted for rate variation with a gamma-distributed rate variation among sites (Γ). In five of the six nuclear orthologs the HKY + Γ model that includes five parameters was preferred. Only in nuclear ortholog C2At1g32130 did GTR + Γ best fit the data.

### Analysis of diploid species

Our strategy was to use the diploid accessions as placeholders in a Bayesian framework to conduct further analyses to show relationships with the polyploids. In a few cases we encountered minor allelic variants from the same accession falling in the same clade in gene trees estimated with RAxML. In these cases we chose as representative the sequence that fell closest to the root of the clade, to limit the number of allele variants for future analysis.

Sequences were edited by Staden package 4.10 [68] and aligned in CLUSTALX 2.0.6 [69], with further manual alignments by MacClade 4.08 OS X [70]. The diploid dataset was imported into BEAUTi (*BEAST 1.6.1 package) to generate the XML format file for *BEAST [71]. Models were selected for each COS on the basis of Akaike Information Criterion by using ModelTest 3.7. “Empirical” base frequencies were used and the Yule speciation process was selected as a prior on the species phylogeny. All MCMC chains were run for 100 million generations with subsampling every 10,000 generations and three independent runs. The three log files were then imported into Tracer 1.5 to get a combined tracer file and to check convergence to the stationary distribution and the effective sample size (ESS) of each parameter. The ESS values were exceeding 200 for all of the tree parameters except for the population size at one node, which had an ESS of 156. The sample files from the three independent runs were combined after discarding their first 10% as burn-in. They were summarized with a greedy consensus in TreeAnnotator 1.6.1 in the BEAST package. The resulting estimated trees (the diploid species tree and the six individuals COS trees) could be viewed in FigTree 1.3.1 in the BEAST package or using the package ape [72] in R [73].

### Analysis of polyploid species

One difficulty with polyploid species is that the placement of allopolyploids or of hybrid species requires adding reticulation events in the species history, which cannot be represented by a bifurcating tree. A network is needed instead. An extra difficulty here is that we do not know which alleles come from the same side of a reticulation event. For a polyploid accession with two alleles at each locus, we can arbitrarily label each allele as “A” or “B” but the “A” alleles do not necessarily share the same parental origin. For instance, it is unknown which of allele “A” or “B” of C2At4g10050 comes from the same parental origin as allele “A” of C2At5g47390. The placement of individual alleles can still be represented in a bifurcating phylogenetic tree, which can provide some evidence about which alleles share the same placement and hence the same parental origin. In our situation however, the extent of conflict between gene trees, even across diploid species, made allele matching difficult and uncertain.

To avoid grouping alleles into putative common parental origins, we analyzed each polyploid allele separately. For each polyploid allele from each locus, we determined where the allele should be placed on the diploid species tree using the fast gene tree/species tree method “NJst,” described in [74]. This method was used instead of *BEAST for two reasons. First, it is fast enough to be repeated once for each of the 823 alleles (and repeated 100 times to obtain bootstrap support values). Second, the NJst method could be modified to apply a subtree constraint. In our case, for each polyploid allele, we constrained the subtree formed by the diploid species to the *BEAST diploid species tree. By doing so, we were able to summarize the results from all polyploid alleles onto the same backbone diploid species tree.

The NJst method uses gene trees, which can include several individuals per species, and estimates a distance matrix between species. The distance between two species is defined as the average number of internodes between the two species, averaged across all gene trees and all pairs of individuals from the two species. Liu and Yu [74] showed that this distance provides a consistent estimate of the bifurcating tree topology under the coalescent model of gene tree discordance. While Saitou and Nei propose using Neighbor-Joining [75] to estimate the species trees based on the internode distance, we used instead the balanced Minimum Evolution (ME) criterion [76] for three reasons: (1) Neighbor Joining greedily aims to minimize the balanced ME criterion [77]. (2) The ME criterion can be evaluated on a set of trees for which a subtree constraint is enforced, whereas Neighbor Joining is an algorithm that builds a tree without any subtree constraint enforced. (3) The ME criterion as implemented in FastME [76] was shown to result in more accurate tree reconstruction than Neighbor Joining [78]. The modified NJst method, called the MEst method, was implemented in R [73] with an external call to fastME [76] for the calculation of the minimum evolution criterion.

For each polyploid allele of each COS, our MEst method was applied to the set of six gene trees as inferred with RAxML, where all polyploid alleles were pruned from the gene trees except for the one allele of interest. The set of candidate species trees consisted of all trees obtained from grafting the polyploidy species onto the *BEAST diploid species tree. The candidate tree with the minimum evolution score was retained and the edge onto which the polyploid species branched off was recorded. If several candidate trees had the same best score, then all these best trees were retained and were given equal weights. For each edge in the diploid species tree, we recorded the number of polyploid alleles whose origin was estimated to be on that edge. To summarize the results at the clade level, we also counted the number of alleles for which the estimated parental origin was within the clade.

In order to account for uncertainty in gene tree estimation, this procedure was repeated 100 times, using gene trees estimated with RAxML from bootstrap sequence alignments. The input to a bootstrap replicate consisted of one bootstrap RAxML tree from each of the six COS. This bootstrap analysis resulted in a sample of size 100 for each edge and for each clade, giving the number of alleles for which the estimated parental origin was on the edge or from within the clade. We summarized each bootstrap sample for each edge and each clade by: (1) the bootstrap support for at least one allele having a parental origin on the edge (or clade), calculated as the number of bootstrap replicates with 1 or more alleles supporting an origin on the edge, (2) the median number of alleles whose parental origin was placed on the edge (or within the clade), (3) a 90% bootstrap confidence interval for the number of alleles whose origin was placed on the edge, calculated by excluding the 5% lowest and 5% highest values in the bootstrap sample. In order to determine if accessions contributed equally to the various estimated parental origin placements, we repeated the procedure by separating out the different accessions for each polyploid species.

## Competing interests

The authors declare that they have no competing interests.

## Authors’ contributions

DS conceived the research and with LM, MB, and YT obtained funding. DC and FR helped design the research. DC performed all the laboratory work from isolating the DNA to generating the DNA sequences to DNA alignment, to data analyses with RAxML and *BEAST. FR designed and screened the primers, developed the SSCP protocol, and advised us on laboratory techniques. CA designed the ME species tree method for polyploids and developed the associated computing pipeline in R. All authors revised several versions of the manuscript. LM generated nuclear orthologs and handled data archiving. All the authors reviewed and approved the final manuscript.
